# Multi-Probe Nano-Genomic Biosensor to Detect *S. aureus* from Magnetically-Extracted Food Samples

**DOI:** 10.3390/bios13060608

**Published:** 2023-06-02

**Authors:** Chelsie Boodoo, Emma Dester, Jeswin David, Vedi Patel, Rabin KC, Evangelyn C. Alocilja

**Affiliations:** 1 Nano-Biosensors Lab, Department of Biosystems and Agricultural Engineering, Michigan State University, East Lansing, MI 48824, USA; boodooch@msu.edu (C.B.); desterem@msu.edu (E.D.); davidje4@msu.edu (J.D.); patelved@msu.edu (V.P.); 2 Global Alliance for Rapid Diagnostics, Michigan State University, East Lansing, MI 48824, USA; 3 Department of Human Biology, Michigan State University, East Lansing, MI 48824, USA; 4 Department of Mechanical Engineering, Michigan State University, East Lansing, MI 48824, USA; 5 Statistical Consulting Center, College of Agriculture and Natural Resources, Michigan State University, East Lansing, MI 48824, USA; kcrabin@msu.edu

**Keywords:** foodborne pathogens, foodborne illness, biosensing, food safety, gold nanoparticles, magnetic nanoparticles

## Abstract

One of the most prevalent causes of foodborne illnesses worldwide is staphylococcal food poisoning. This study aimed to provide a robust method to extract the bacteria *Staphylococcus aureus* from food samples using glycan-coated magnetic nanoparticles (MNPs). Then, a cost-effective multi-probe genomic biosensor was designed to detect the nuc gene of *S. aureus* rapidly in different food matrices. This biosensor utilized gold nanoparticles and two DNA oligonucleotide probes combined to produce a plasmonic/colorimetric response to inform users if the sample was positive for *S. aureus*. In addition, the specificity and sensitivity of the biosensor were determined. For the specificity trials, the *S. aureus* biosensor was compared with the extracted DNA of *Escherichia coli*, *Salmonella enterica* serovar Enteritidis (SE), and *Bacillus cereus.* The sensitivity tests showed that the biosensor could detect as low as 2.5 ng/µL of the target DNA with a linear range of up to 20 ng/µL of DNA. With further research, this simple and cost-effective biosensor can rapidly identify foodborne pathogens from large-volume samples.

## 1. Introduction

According to estimates, in 2010, 600 million individuals (almost 1 in 10) worldwide contracted a disease by consuming food infected with microorganisms, which led to 420,000 fatalities [1]. One of the most prevalent foodborne illnesses worldwide is staphylococcal food poisoning (SFP). Enterotoxigenic strains of *Staphylococcus aureus* and other species are the principal producers of staphylococcal enterotoxins, which cause SFP [2]. The bacteria *Staphylococcus* is spherical, nonsporulating, and non-motile. Under a microscope, it can form in pairs, short chains, or clusters resembling grapes [3]. SFP can occur for a variety of reasons. Poor hygiene procedures during food processing, cooking, or distribution are the leading causes of SFP outbreaks [4,5]. Additionally, after contamination, improper refrigeration of foods can promote the growth of *Staphylococcus* and/or the synthesis of toxins, leading to food poisoning [6].

These dangerous bacterial species must be detected before they reach the consumer to avoid infection, disease, and even death. Detecting food pathogens is complicated by the need to isolate and concentrate bacteria from various food matrices. It is difficult to separate the pathogens from the food matrix, remove the inhibitory compounds, and recover the bacteria without disrupting the cell viability [7]. These harmful bacteria can be found using conventional techniques successfully. However, the results for the conventional recommended culture-based technique can take 4–7 days to be conclusive [8,9]. Numerous efforts have been made to increase bacterial concentration as a result, including those involving biofilms [10], centrifugation [11], dielectrophoresis [12], filtration [13], and magnetic nanoparticles (MNPs) [14,15,16]. These more recent methods have reduced the time from days to hours. However, they require skilled personnel to conduct the assays and operate the equipment.

Furthermore, sample preparation is required, and the materials/equipment needed are expensive, which limits these technologies to developed countries. Biosensors can be used to detect foodborne pathogens such as S. aureus rapidly. Biotechnology has had an increased interest in biosensors because they are easy to use, portable and provide a rapid response within minutes [17]. Antibody-based immunoassay biosensors have been used for detecting *S. aureus*. However, the conjugation and purification processes are time-consuming. The antibodies are also high-cost, and the instruments are expensive [18]. Table 1 summarizes different biosensor technologies that have been developed to detect *S. aureus* based on their transducer, bio-recognition element, preparation, sample tested, assay time, and limit of detection (LOD).

MNPs with glycan coatings can separate *S. aureus* from food matrices. The glycans on the surface of the nanoparticles have electrostatic interactions with the proteins of the bacteria, which allows the MNPs to capture S. aureus through separation [26] magnetically. For the concentration and detection of many bacterial species implicated in foodborne pathogens, such as *S. aureus*, glycan-coated MNPs have been used [27]. This differs from the often-employed antibody-antigen MNP techniques, which require refrigeration [28,29,30]. In addition, the glycan-coated MNPs are more cost-effective than IMS, require short incubation times of 5 min, and can bind to proteins on the bacterial surface [26]. We concentrated *S. aureus* using MNPs coated in glycan chitosan. The MNPs were synthesized using the procedure in [31,32]. As confirmed through TEM, the MNPs are 99 ± 58 nm.

Conventional bio-detection assays utilize polymerase chain reaction (PCR), fluorescence, or luminescence. However, these techniques require costly materials; luminescence can have high background noise [33], and fluorescence requires the analyte to be less than 15 nm which results in limited analyte detection and issues detecting low volumes of the pathogen [34]. PCR can be extremely sensitive, where minor contaminants in the samples can lead to misdiagnosis [35]. Biosensors can be categorized based on the element used in biological recognition and the transducer. The biological element can be enzyme, antibody or DNA-based [36]. The transducer can be electrochemical, optical, colorimetric, mass, or magnetic. In this paper, the biosensor is DNA-based and plasmonic [37]. We use a gold nanoparticle (GNP) based biosensor to detect *S. aureus*, specifically the thermonuclease (nuc) gene. Nuc is an active, surface-localized enzyme in *S. aureus* [38,39,40,41] We hypothesize that targeting nuc with our GNP biosensor can detect *S. aureus* after concentrating it with the MNPs.

The GNPs are red due to their localized surface plasmon resonance (LSPR) around 20–30 nm [42]. The wavelength for the absorption peak is 520 nm for red, which we can observe in the Nanodrop One-C spectrophotometer. Usually, the GNPs are not aggregated because of electrostatic repulsion. However, the GNPs developed in this laboratory aggregate upon protonation from an acid when the target DNA is absent. This leads to GNPs having a color change from red to purple/blue, resulting in an absorption wavelength shift to the right, away from 520 nm. When the acid (in our case, 0.1 M hydrochloric acid) is added, the GNP-oligonucleotide complex hybridizes with the target DNA if present. The target DNA protects the GNPs from aggregating and keeps the color red.

This work is novel because two oligonucleotide probes 30 base pairs each were used to detect the *S. aureus* nuc gene, and there was no amplification of *S. aureus* due to the MNPs concentrating the pathogen. GNP is thiolated during synthesis, so we do not need further probe functionalization. The GNP-based biosensor only needs the dextrin-capped GNPs, the two oligonucleotide probes specific to the *S. aureus* nuc gene, and the sample for testing. This biosensor is rapid and low cost, so it can be used often to test food samples for *S. aureus*.

## 2. Materials and Methods

Frozen bacterial stock cultures of *S. aureus*, *E. coli* O157, *S.* Enteritidis, and *B. cereus* were obtained from the Nano-Biosensors Laboratory at Michigan State University (MSU). Glycan-coated MNPs were used from the Nano-Biosensors Lab, MSU. These MNPs were developed beforehand and used in previous papers [43,44,45]. For the food extraction, Whirl-Pak bags (92 oz.) were purchased from VWR International (Radnor, PA, USA). Fleximag Separators were purchased from Spherotech Inc (Lake Forest, IL, USA).

CHROMagar *S. aureus* was purchased from CHROMagar (Paris, France). Phosphate Buffer Solution (PBS) pH 7.4, Hydrochloric acid (HCl) (ACS reagent, 37%), gold (III) chloride trihydrate (HAuC_4_), sodium carbonate (Na_2_CO_3_), 11-mercaptoundecanoic acid (MUDA) (HS(CH_2_)_10_CO_2_H), Tryptic Soy Agar (TSA), Tryptic Soy Broth (TSB), sodium dodecyl sulfate (SDS) (C_12_H_25_NAO_4_S), and dextrin from potato starch (C_6_H_12_O_6_) were purchased from Sigma Aldrich (St. Louis, MO, USA). TSA, TSB, and PBS were prepared as directed by the supplier, Sigma Aldrich. The HCl was diluted with ddH2O to make 0.1 M. The Powerlyzer Microbial Kit and AE buffer solution used for DNA extraction were purchased from Qiagen (Germantown, MD, USA). In addition, a NanoDrop One-C from ThermoFisher Scientific was used to quantify the DNA concentrations and absorbance spectra data (Waltham, MA, USA). The device has a working spectral range of 190–850 nm and wavelength accuracy of ±1 nm, with complete specifications detailed online and in the user manual [46,47].

### 2.1. Bacterial Culture

Frozen stock cultures of each bacterial species were kept at −80 °C. Master plates were made by streaking 10 µL of a stock culture on TSA and incubating at 37 °C for 24–48 h. Before being replaced, these plates were stored at 4 °C for four weeks. Genomic DNA was extracted using the Qiagen Powerlyzer DNA extraction kit. Colonies were transferred into 9 mL of TSB and incubated overnight, with 1.8 mL of the resulting transfer used for each DNA collection tube. The concentration for the extracted DNA was measured on the Nanodrop One-C using the dsDNA setting. The samples were diluted to the set concentration needed for specificity and sensitivity testing.

### 2.2. Magnetic Nanoparticles for Extraction

**Figure 1:**
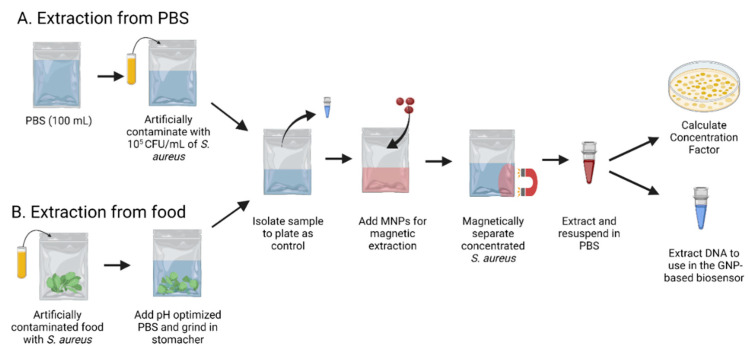
Using magnetic nanoparticles to extract *S. aureus* from (**A**) PBS and (**B**) food.

Magnetic Extraction in PBS. For bacterial inoculation, 4-h spiked bacterial cultures were serially diluted to 10^−3^, which had an equivalent of 1.9 × 10^5^ CFU/mL after plating. 1 mL of the *S. aureus* was then immediately transferred to a Whirl-Pak bag containing 101 mL of PBS and mixed, as depicted in Figure 1A. A control sample of 1 mL was taken from the bag to be plated before the magnetic extraction step. After that, 1 mL of MNPs was added and stirred in the Whirl-Pak bag. Then it was incubated for 5 min at room temperature. A magnetic rack was then fastened to the Whirl-Pak bag for 5 min to allow the MNPs to bind to the *S. aureus*. Since the MNPs are positively charged with the glycan chitosan coating, and *S. aureus* has a negative surface charge [48] which causes electrostatic attraction. After, the supernatant was removed, and the remaining sample with MNPs was resuspended in 1 mL of sterile PBS. That suspension was used as the concentrated sample. The ultimate bacterial concentration was ascertained by serially diluting the sample and plating it on selective media. Before analysis, all plates were incubated at 37 °C for 24 h.

The bacterial concentration was accomplished through colony counting of plates with 20–300 individual colony-forming units (CFUs). The concentration factor was calculated using the following formula:

(1) $$Concentration\;Factor=\frac{CFUs\;in\;treated\;sample}{CFUs\;in\;control}$$

Magnetic Extraction in Food. Four food matrices were selected based on recent multistate outbreak data [49]. The procedure was also described in a previous paper published in this laboratory [50]. The protocol begins with artificial contamination, shown in Figure 1B. The overall method is detailed in Figure 1. First, 25 g of the food matrix were weighed in a Whirl-Pak bag. Following artificial contamination of food samples, the bacteria were given an hour at room temperature to adapt. After this incubation, each sample had 225 mL of PBS added in sterile Whirl-Pak bags before being put into a stomacher for 2 min. Next, the original bag of the liquified food matrix was removed, and two Whirl-Pak bags containing 100 mL of liquified food each were prepared. One bag was labeled as the control and plated to find the initial bacterial concentration. Next, magnetic extraction was performed on the other bag, which had been labeled as the treatment.

Like PBS tests, 1 mL of MNPs was poured into the Whirl-Pak bag, stirred, and let sit for 5 min at room temperature. After that, a magnetic rack was fastened to the Whirl-Pak bag. After five more minutes, the supernatant was removed using a pipet, and the remaining material was resuspended in 1 mL PBS. The final sample was serially diluted and plated on selective media to find the ultimate bacterial concentration. For each food matrix, studies were carried out in triplicate, and all plates were incubated at 37 °C for 24 h before analysis. The target bacterial colonies and any atypical microflora were distinguished according to the manufacturer’s recommendations for selective media.

### 2.3. Oligonucleotide Probe Designs

Two oligonucleotide probes were explicitly designed to detect the *S. aureus* nuc gene, with a genome size of approximately 2.82 Mb [51]. Both probes targeted the nuc gene with the following sequences: TGTTTCGAAAGGGCAATACGCAAAGAGGTT and TAGCTCAGCAGATGCATCACAAACAGATAA. The probes were designed using NCBI BLAST (National Center for Biotechnology Information Basic Location Alignment Search Tool), where the specificity was confirmed. The sequences for the probes were chosen based on their lengths, and they had the most specificity to the nuc region of *S. aureus*, which is composed of 655 bp. If 3 to 6 nucleotide sequences are changed in the oligonucleotide probe, the biosensor could lose some of its specificity. The probes were purchased with 5′ amination from Integrated DNA Technologies (IDT) (Coralville, IA, USA). Two probes were used to cover two regions in the *S. aureus* nuc gene sequence.

### 2.4. GNP Synthesis and Surface Coating

**Figure 2:**
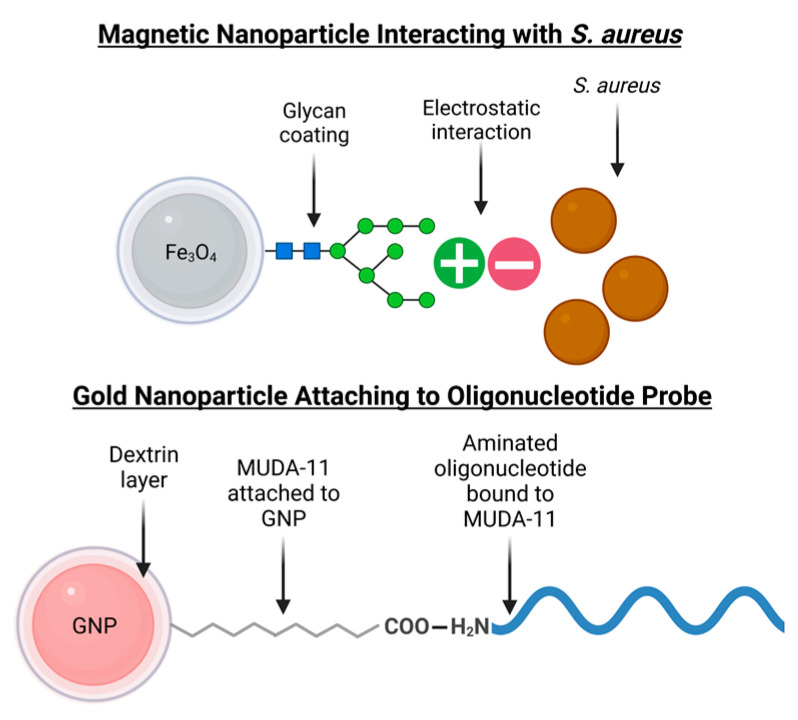
The MNP is made with Fe_3_O_4_ and coated with glycan chitosan. The chitosan glycan is positive, and the surface of the *S. aureus* is negative. They are attracted by electrostatic interaction. The GNP is capped with dextrin that is attached to MUDA-11. The COO- group in MUDA is noncovalently bound to the amine group in the oligonucleotide probe.

Dextrin-capped GNPs were synthesized using the methods from Yrad et al. [52]. In this procedure, 5 mL of 20 mM of HAuCl_4_ was added dropwise to 20 mL of freshly made 25 g/L dextrin. 10% Sodium carbonate (Na_2_CO_3_) was added to bring the pH to 9.0. The mixture was incubated at 150 °C until the GNPs were 20–30 nm, which was confirmed using transmission electron microscopy (TEM). The extinction coefficient was used by Jin et al. [53]. After the GNPs were cooled to room temperature, they were coated with thiols using MUDA and resuspended in 500 µL borate buffer. The way the dextrin-capped GNPs binds to the oligonucleotide probe is demonstrated in Figure 2.

### 2.5. Biosensor Design and Optimization

The probes were stored at −20 °C and diluted to 25 μM with IDT TE pH 8 buffer. For every experiment, the DNA samples were 10 μL each. The GNPs were 5 μL, and both probes were 2.5 μL totaling 5 μL for the probes. For the specificity tests, 5 PCR tubes were used per trial; one tube was the control with nuclease-free water, another with the target *S. aureus* DNA, and one each for the nontarget DNA: *E. coli* O157, *S.* Enteritidis, and *B. cereus*. All DNA samples were 20 ng/µL for the specificity experiments. For sensitivity experiments, the *S. aureus* DNA was serially diluted from 20 to 10, 5, and 2.5 ng/µL, where 2.5 ng/µL was the lowest concentration that could be detected.

The tubes were properly pipette mixed before heating in the thermocycler for one cycle of denaturation at 95 °C for 5 min, annealing at 55 °C for 10 min, and cooling at 25 °C. After the denaturation, the tubes were put on a rack to cool to room temperature.

During testing, absorbance measurements and images were collected after the HCl application at the predetermined optimized time. The optimized amount of HCl was determined by observing the color change due to the aggregation of the GNPs when there is an absence of the target DNA. The designed oligonucleotide probes conjugated with the GNPs through a thiol-amine interaction. The oligonucleotide was aminated, binding to the thiol on the GNPs. Therefore, this stable complex could bind to the target DNA through the oligonucleotide probe specific to the target *S. aureus* DNA sequence.

**Figure 3:**
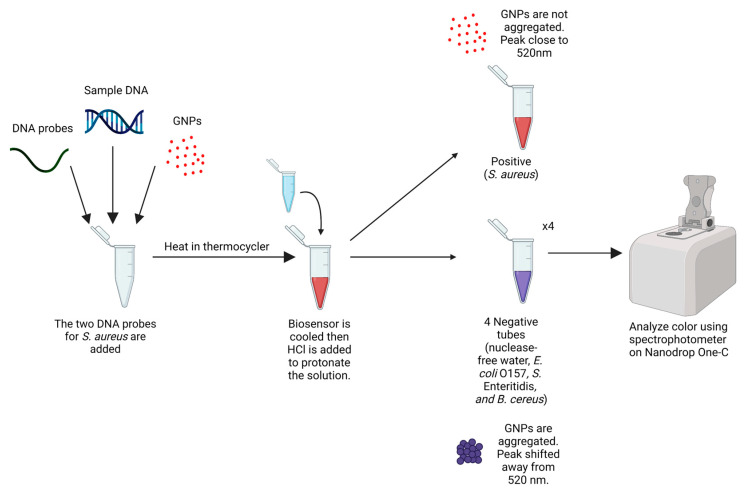
Protocol to create and interpret the GNP biosensor.

The time between the HCl addition and the readings in the spectrophotometer was optimized to be between 1–10 min due to the color changes of the control and nontarget tubes. As shown in Figure 3, the color change was confirmed in a Nanodrop One-C spectrophotometer. The red color from the positive tubes had a peak wavelength at 520 nm, and the negative tubes had a peak shifted to the right, away from 520 nm. Results were analyzed through the generation and inspection of absorbance spectra on MATLAB.

**Figure 4:**
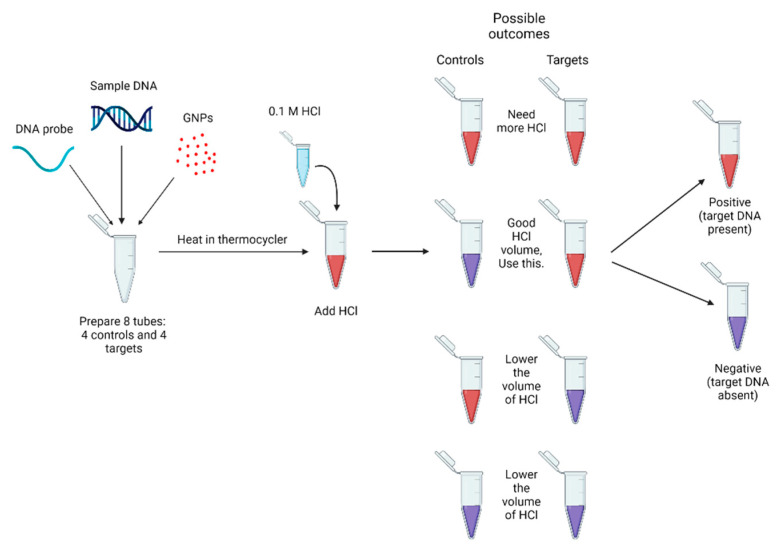
Optimizing the GNP biosensor to find the ideal volume of HCl needed.

To use the GNP biosensor, the correct volume of HCl must be known. As shown in Figure 4, eight tubes are used to optimise it, where 4 are targets and 4 are controls. The volume of 0.1 M HCl is varied to see if there are color changes. The desired result is the control blue/purple and the target red. If the control is red, the biosensor needs to have more volume of 0.1 M HCl added. If the target is blue/purple, less 0.1 M HCl must be added.

### 2.6. Statistical Analyses

Results were analyzed using RStudio (version 2022.07.2). To detect *S. aureus* from various food matrices, an experiment was set up in a randomized complete block design with seven treatments (different foods with control) and replicated seven times. To test the effect of treatments on the response (peak shifts from 520 nm), data were subjected to linear mixed effects ANOVAs using the package ‘*lmerTest’*. Treatments were modeled as fixed effects, and replicates were modeled as random effects. Model residual diagnostics were performed visually to check for assumptions. The model fitted versus residual plot was used to assess constant variance, and QQ plots to assess normality. Outliers were detected based on the Bonferroni outlier test from the ‘*car*’ package and mean-shifting outliers with absolute studentized residuals greater than three were removed from the model. Data were log-transformed to meet normality and constant variance assumptions. A post hoc mean separation test was conducted using the ‘*emmeans*’ package. The response and standard errors were back-transformed, and estimated marginal means were reported.

For assessing the sensitivity of the *S. aureus* GNP biosensor, a separate experiment was set up in a randomized complete block design with six treatments (different concentrations of target and control) and was replicated nine times. Similar to the food analysis, a linear mixed effects ANOVA was employed to test the fixed effects of treatments on response. Treatments were modeled as fixed effects, and replicates were modeled as random effects. Model residual diagnostics were performed visually to check for assumptions. The model fitted versus residual plot was used to assess constant variance, and QQ plots to assess normality. Outliers were detected based on the Bonferroni outlier test from the ‘*car*’ package and mean-shifting outliers with absolute studentized residuals more significant than three were removed from the model. The post hoc mean separation test was conducted using the ‘*emmeans*’ package, and the estimated marginal means were reported.

For the specificity analysis, a randomized complete block design was employed with five treatments and measurements taken in two periods and replicated nine times. A linear mixed effects ANOVA was used to test the effects of treatments on the response. The considered fixed effects were treatments, time, and their interactions. Replicates and replicate × time interaction were considered random effects. Model residual diagnostics were performed visually to check for assumptions. The model fitted versus residual plot was used to assess constant variance, and QQ plots to assess normality. Outliers were detected based on the Bonferroni outlier test from the ‘*car*’ package and mean-shifting outliers with absolute studentized residuals greater than three were removed from the model. The post hoc mean separation test was conducted using the ‘*emmeans*’ package, and the estimated marginal means were reported.

## 3. Results

### 3.1. Concentration Factor for the Extraction of S. aureus in Food and PBS

Using the glycan-coated MNPs, *S. aureus* was successfully extracted from milk, sausage, deli ham, and romaine, as evidenced by the growth of viable colonies in selective agar plates. In addition, this *S. aureus* biosensor was tested with the DNA extracted from artificially contaminated food samples of sausage, romaine lettuce, ham, and milk. The concentration factor for extracting the DNA from PBS versus the food matrices is shown in Table 2. The concentration factor was calculated using Equation (1). The control PBS where MNPs were not used had a lower concentration than the treated samples where MNPs were used.

The CF for the extraction from PBS is much higher than that of the food matrices. This can be due to the food particles being barriers for the MNPs to bind S. aureus. The natural micro-flora/components of the food matrices are increased with the MNPs, and may also impact the de-creased CF. The different pH levels have been shown to impact the zeta potential of S. aureus with MNPs [32]. The MNPs have an increased electrostatic attraction at a low pH because the amino group from the chitosan is protonated [54]. The different food samples can cause changes in the pH which can impact the capture efficiency of S. aureus with the MNPs. However, if there are too many negative ions, the MNPs can become negatively charged or neutralized [55]. The CF for the extraction from PBS is much higher than that of the food matrices. This can be due to the food particles being barriers for the MNPs to bind *S. aureus*. The natural micro-flora/components of the food matrices are increased with the MNPs, and may also impact the de-creased CF. The different pH levels have been shown to affect the zeta potential of *S. aureus* with MNPs [32]. The MNPs have an increased electrostatic attraction at a low pH because the amino group from the chitosan is protonated [54]. The different food samples can cause changes in the pH which can impact the capture efficiency of S. aureus with the MNPs. However, if there are too many negative ions, the MNPs can become negatively charged or neutralized [55].

### 3.2. Peak Shifts and Color Change for GNP Biosensor

**Figure 5:**
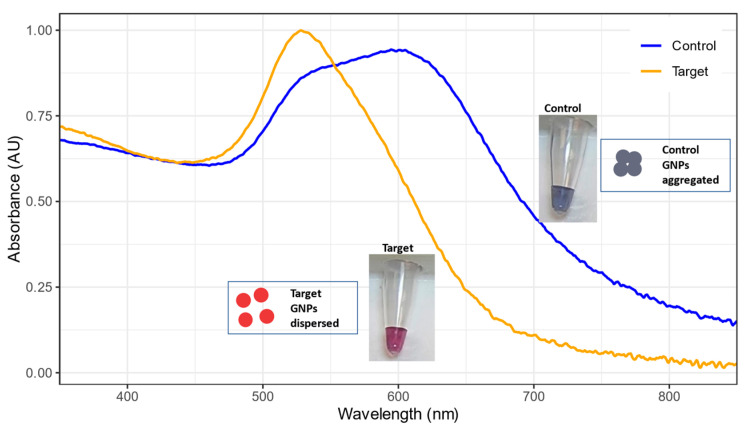
Spectra of the control (nuclease-free water) versus the target sample (*S. aureus* at 20 ng/µL). Next to the spectra are the tubes showing the colors as a reference. Above is a schematic of the GNPs.

GNP-based biosensors developed in the Nano-Biosensors Laboratory have been used to detect various bacteria [36,50,56,57,58,59,60,61] and viruses [52,62]. When we use the GNP-based biosensor, we can observe that the peak is closer to 520 nm for the target samples and shifted to the right from 520 nm for the controls (Figure 5). As shown at the top of Figure 5, the aggregation of the nanoparticles with the sample that does not have the target DNA causes the peak shift. The color of the tubes is also shown for reference in Figure 5. The target sample has the *S. aureus* target DNA at 20 ng/µL, which is why the tube is redder than the control, and the peak is much closer to 520 nm than the control.

### 3.3. Detecting S. aureus from Various Food Matrices

According to the FDA [63], *S. aureus* will cause food poisoning at 5.0 × 10^5^–1.0 × 10^6^ CFU/g. Therefore, the biosensor was tested to detect levels below this amount. The CFU/g of *S. aureus* for milk was 237. For romaine lettuce, it was 13.1. For ham, it was 201. For sausage, it was 132. Our CFU/g concentration was calculated by plating the inoculation concentration for each experiment. To determine the pathogen concentration, a Nanodrop One-C was used. However, the concentration of the DNA may not all be *S. aureus* because the DNA may also have some of the microflora from the food matrices.

**Figure 6:**
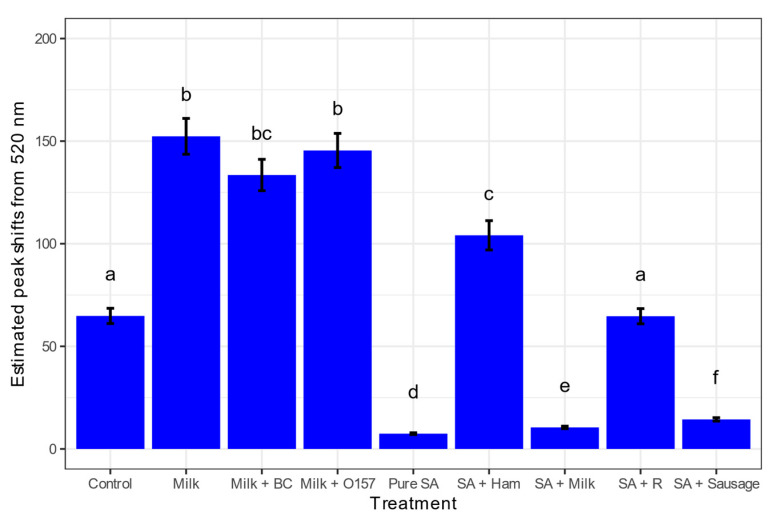
Estimated marginal mean peak shifts away from 520 nm from various food matrices used in the study after 5 min. Error bars on the top of each bar represented SEM (*n* = 9). Treatments with different letters on the top of the bars are significantly different at α = 0.05. All samples were diluted to 20 ng/µL. From left to right: control with nuclease-free water, pure milk without artificial contamination, milk with *B. cereus*, milk with *E. coli* O157, pure *S. aureus* (SA), SA with ham, SA with milk, SA with romaine lettuce, and SA with sausage.

**Figure 7:**
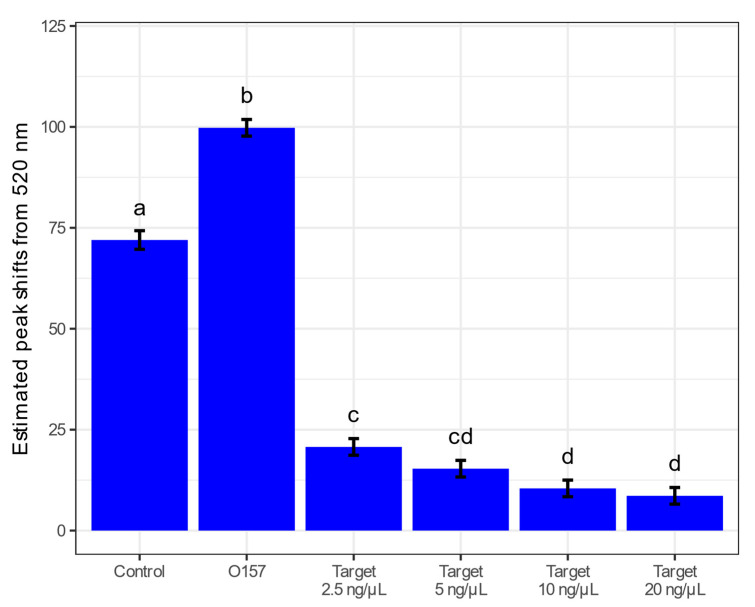
Estimated marginal mean peak shifts away from 520 nm at 5 min. Left to right shows the control and *E. coli* O157, then the different concentrations of target samples of *S. aureus* at 2.5, 5, 10, and 20 ng/µL. Error bars on the top of each bar represent SEM (*n* = 9). Treatments with different letters on the top of the bars are significantly different at α = 0.05.

After the artificial contamination of foods, magnetically extracted bacterial cells were grown for 6 h, and the DNA was extracted. The difference in peak shifts from 520 nm is displayed in Figure 6 and Figure 7. The undiluted DNA samples were romaine with *S. aureus* 46 ng/µL, sausage with *S. aureus* 199 ng/µL, ham with *S. aureus* 135 ng/µL, and milk with *S. aureus* 302 ng/µL. The lowest initial DNA concentration was milk without artificial inoculation at 20 ng/µL, so all the samples were diluted to that concentration. The non-target samples were milk without artificial contamination, with *E. coli* O157, and with *B. cereus*.

The control with water served to check for any contamination in the system. Compared to the negative nontarget milk, *S. aureus* was successfully detected in all food matrices, milk, ham, sausage, and romaine lettuce. This negative nontarget included DNA from the natural microflora, representing a more realistic scenario. During the DNA extraction process, particles of ham were visible, which may have interfered with the quality of the extracted DNA. Romaine lettuce may not have had a low peak shift from 520 nm with the GNP-based biosensor because of the natural microflora. The CF for the nonselective TSA plates with romaine lettuce was 2.27 versus for the selective plates a CF of 0.86. Romaine lettuce had the biggest difference in CF between the selective and nonselective media. The results are statistically shown in Figure S1.

### 3.4. Sensitivity of the S. aureus GNP Biosensor

To ascertain the sensitivity of the biosensor, *S. aureus* was tested at various concentrations. The estimated mean peak shifts from 520 nm had a significant decreasing trend, where mean peak shifts away from 520 nm were highest when the concentration was lowest (2.5 ng/µL). In this experiment, 2.5 ng/µL was the lowest concentration the biosensor could detect. (Figure 7 and Figure S2). The mean peak shifts from 520 nm were significantly higher for control and O157 (Figure 7). The lower the concentration of *S. aureus*, the larger the peak shift is away from 520 nm.

### 3.5. Specificity of the S. aureus GNP Biosensor

**Figure 8:**
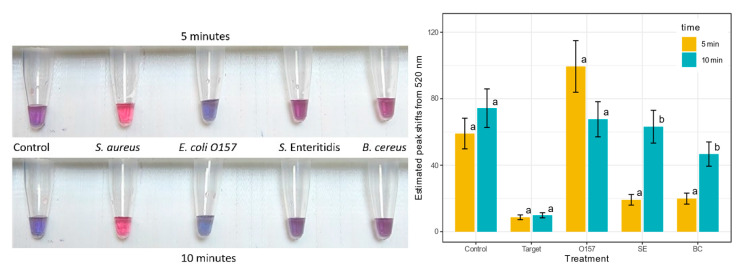
Visible results for the specificity of the *S. aureus* GNP biosensor after 5 min of protonation on the top left panel and 10 min on the bottom left panel. The tubes (from left to right) are the control with nuclease-free water, target *S. aureus*, nontargets: *E. coli* O157 (O157), *S.* Enteritidis (SE), *B. cereus* (BC), all at 20 ng/µL. The right graph shows the difference in peak shifts away from 520 nm for specificity at 5 and 10 min. Error bars on the top of each graph represent SEM (*n* = 9). Within each time (5 min and 10 min), different letters on the top of the bars significantly differ at α = 0.05. The higher the peak shift, the more aggregated the biosensor is, which indicates that the sample is negative. Treatments with different letters on the top of the bars are significantly different at α = 0.05.

To determine if the biosensor was specific to *S. aureus*, the DNA of other foodborne pathogens was used: *E. coli* O157, *S.* Enteritidis, and *B. cereus*. All the samples were diluted to the same concentration of 20 ng/µL. Figure 8 has a visual representation of the tubes with their color changes at 5 and 10 min and the difference in peak shifts for each sample. These samples were measured until 10 min because the *S.* Enteritidis and the *B. cereus* samples took longer to aggregate than the *E. coli* O157 and the control of nuclease-free water. After 10 min, the target *S. aureus* was still red, and the negative samples had aggregated to turn purple/blue. Therefore, the biosensor is specific to *S. aureus*, as shown by the significantly small peak shifts from 520 nm for the target samples compared to other samples in the experiment (Figure 8, right panel).

## 4. Discussion

A biosensor to rapidly detect *S. aureus* in food matrices can decrease the chances of illnesses and an outbreak, especially in low-resource settings. Many current methods of detection for *S. aureus*, such as antibody-antigen reactions, ELISA, PCR, and IMS, are expensive and require specific expensive equipment and trained personnel to be used. This work used glycan-coated MNPs to concentrate *S. aureus* from PBS and food matrices. Then the DNA from the concentrated samples were detected using the GNP-based biosensor. This biosensor detected 2.5 ng/µL of *S. aureus* without any amplification. However, the MNPs resulted in a much higher CF for the PBS tests versus the food samples. More investigation needs to be done into the cause of this. It could be due to the food particles blocking the MNPs from getting to the *S. aureus*, or the components of the foods may be interfering with the capture efficiency of the MNPs. Furthermore, a multiplex biosensor can be created to detect other foodborne pathogens. For example, a biosensor using the GNP-based biosensor technology from our laboratory was recently developed for *E. coli* O157.

This biosensor can be improved to detect *S. aureus* from other food matrices and utilized as a low-cost, rapid biosensor for *S. aureus* that would not need laboratory facilities. Though it would be more costly, further purification of the DNA samples with ham may lead to a lower peak shift from 520 nm to detect *S. aureus*. The GNP biosensor is a cost-effective alternative to conventional methods like PCR and IMS, and it does not need expensive equipment. If a thermocycler is not accessible for denaturing and annealing the GNPs, probes, and DNA, a water bath at 95 °C for 5 min and another at 55 °C for 10 min, or a programmable water bath can be used because the biosensor does not use amplification. If one does not have access to a Nanodrop One-C, the color can be observed visually. It has been shown that a smartphone can be used to determine the result of a colorimetric biosensor [63]. Hence, a picture can be taken, and an eyedropper tool on the computer or smartphone can be used to determine and quantify the colors of the tubes.

## 5. Conclusions

The glycan-coated MNPs could extract and concentrate *S. aureus* from PBS and food samples. Results showed that the *S. aureus* GNP biosensor was specific *to S. aureus* and could detect the *S. aureus* DNA as low as 2.5 ng/µL. When compared to the negative nontarget, successful detection of *S. aureus* was also completed in the sausage, ham, romaine lettuce and milk food samples. This GNP-based biosensor is a rapid and cheap alternative to conventional methods, such as PCR, to detect *S. aureus*. Due to its low cost, it can be implemented in low-income countries. Since the GNPs do not require much functionalization, they do not need any preparation before use. In the future, this biosensor can be improved by increasing the CF of the MNPs and detecting *S. aureus* in food samples. More food samples can also be tested to investigate the effects of food matrices with the CF and detection. In the future, if this biosensor works with a phone application, the Nanodrop will not be needed, which can lower the cost of testing. With these improvements, this biosensor can be rapid, low-cost, and accessible to help detect *S. aureus* in food matrices.

## Figures and Tables

**Table 1:** Summary of different biosensor technologies developed to detect *S. aureus*.

Transducer	Bio-Recognition Element	Preparation	Sample(s)	Assay Time	LOD	Source
Impedimetric	Peptide	Fabrication (<2 h)	Pure culture	30 min	102	[19]
Piezoelectric	Aptamer	Gold electrode (7 h)	Milk and pure culture	1 h	4.1 × 10^1^, 41 CFU/mL	[20]
Piezoelectric	Antibody	Au electrode fabrication (1 day)	Milk	2 h	6.1 ng/mL	[21]
Colorimetric	Aptamer	Nanobead synthesis (1 day)	Milk, lettuce, turkey sausage, ground beef	40 min	40 CFU/mL	[22]
Fluorescence	Aptamer	Spike samples, PEI-MNP separation, extraction (30 min)	Skim milk	1 h	2.9 × 10^2^ CFU/mL	[23]
LSPR	Antibody	GNP preparation (3 h)	Pure culture	1 min	120 CFU/mL	[24]
SPR direct detection; SPR sandwich assay	Antibody	SPR sensor chip (2 days), incubate *S. aureus* 10 min	Buffer; Buffer, Milk	~1 h	5 ng/mL; 0.5 ng/mL	[25]

**Table 2:** The average concentration factor and associated standard errors (*n* = 3) of *S. aureus* were extracted from food matrices and PBS. The samples used MNPs to extract *S. aureus*.

Sample	Concentration Factor	Standard Error
PBS	65.5	8.87
Milk	2.46	0.61
Romaine Lettuce	2.27	0.12
Deli Ham	1.38	0.19
Sausage	1.54	0.22

## Data Availability

The data presented in this study are available upon request from the corresponding author.

## Supplementary Materials

The following supporting information can be downloaded at: https://www.mdpi.com/article/10.3390/bios13060608/s1, Table S1: Significance of fixed effects and associated F values for the experiments conducted. Figure S1: Spectra to show the sensitivity of the GNP biosensor 5 min after HCl 0.1 M is added to protonate the GNPs for nine replicates.
