# Supplementary material for: Multi-Probe Nano-Genomic Biosensor to Detect S. aureus from Magnetically-Extracted Food Samples
Source: Biosensors (Basel). 2023 Jun 2;13(6):608. doi: 10.3390/bios13060608 (PMC10295863; doi:10.3390/bios13060608)
Supplement: Supplementary file 1 [file biosensors-13-00608-s001.zip › biosensors-2306441-supplementary.pdf]

Supporting Information

Multi-Probe Nano-Genomic Biosensor to Detect *S. aureus* from Magnetically-Extracted Food Samples

Chelsie Boodoo, Emma Dester, Jeswin David, Vedi Patel, Rabin KC and Evangelyn C. Alocilja *

Table S1. Significance of fixed effects (P > F) and associated F statistic on treatments for the three experiments conducted. Within each experiment, p values followed by *** are significantly different at 0.001 alpha level. For food and sensitivity experiments, treatments were considered fixed effects; for specificity experiments, treatment, time, and treatment x time interaction were considered fixed effects.

Fixed Effects	Experiment					
	Food		Sensitivity		Specificity	
	F Statistic	*p*-Value	F Statistic	*p*-Value	F Statistic	*p*-Value
Treatment	428.2	<0.001 ***	435.9	<0.001 ***	175.7	<0.001 ***
Time	--	--	--	--	78.7	<0.001 ***
Time x Treatment	--	--	--	--	11.4	<0.001 ***

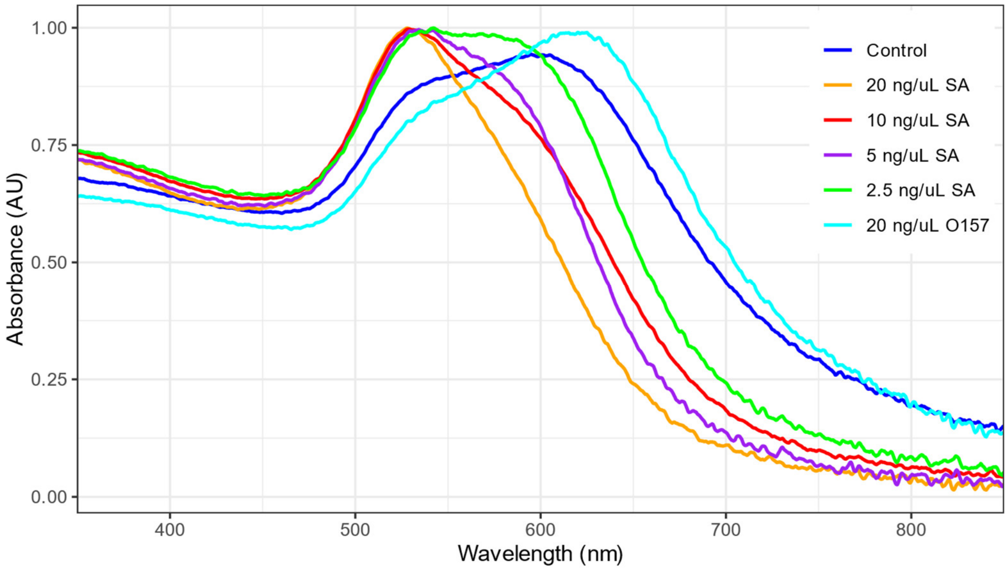

Figure S1. Spectra shows the sensitivity of the GNP biosensor 5 minutes after HCl 0.1 M is added to protonate the GNPs for 9 replicates. The control is nuclease-free water; the nontarget is *E. coli* O157 at 20 ng/μL. The target samples of *S. aureus* are 20, 10, 5, and 2.5 ng/μL.
